# A novel method for efficient generation of antigen-specific effector T-cells using dendritic cells transduced with recombinant adeno-associated virus and p38 kinase blockade

**DOI:** 10.1186/s12967-019-02163-4

**Published:** 2019-12-19

**Authors:** Leonardo Mirandola, Maurizio Chiriva-Internati, Robert Bresalier, Lucia Piccotti, Fabio Grizzi, Francesco M. Marincola

**Affiliations:** 1 Kiromic, Inc, 7707 Fannin St., Suite 140, Houston, TX 77054 USA; 2 Division of Internal Medicine, Department of Gastroenterology Hepatology and Nutrition, The University of Texas MD Anderson Cancer Center, 1515 Holcombe Blvd, Houston, TX 77030 USA; 3 Department of Immunology and Inflammation, Humanitas Clinical and Research Center, Via Manzoni 56, 20089 Rozzano, Milan, Italy

**Keywords:** Ovarian cancer vaccine, Dendritic cells, p38 MAP kinase, T-regs

## Abstract

**Background:**

The inefficacy of standard therapeutic strategies for ovarian cancer is reflected by the enduring poor prognosis of this malignancy. Due to the potential for exquisite specificity, sensitivity and long-term memory, immunotherapy offers an alternative modality for durable control of the disease, provided appropriate antigens can be identified and presented in the right context.

**Methods:**

We tested a novel dendritic cell vaccine formulation to reprogram autologous antigen-specific T-cells in vitro, in vivo in a murine model of ovarian cancer, and ex vivo using human cells from patients.

**Results:**

We show that dendritic cells (DCs) treated with a p38 MAPK inhibitor and transduced with a recombinant adenovirus associated vector (AAV) expressing Sperm protein (Sp) 17 are highly effective in generating antigen-specific T-cell cytotoxic response against ovarian cancer cells. Additionally, these DCs enhanced the differentiation of effector T-cells while reducing the frequency of Foxp3^+^ T-reg cells in vitro.

**Conclusions:**

This work provides a rationale for translation of pharmacologically reprogrammed DCs into clinical trials for prevention of tumor recurrence and progression in high-risk ovarian cancer patients.

## Background

Given the high mortality rate and recurrence risk in ovarian cancer (OC) patients, the current challenge is to develop new therapeutic strategies to prevent disease relapse and progression [1]. One of the most promising approaches to accomplish these goals is to induce the patient’s immune system to direct a specific and durable anti-tumor response. The potential of immune surveillance in OC is supported by the observation that the presence of tumor-infiltrating lymphocytes (TILs) correlates with a favorable prognosis [2]. TIL evaluation has consistently proven useful for surgical decision-making in OC treatment [3]. Unfortunately, spontaneous immune reactions against tumor cells are ineffective due to multiple immunological evasion mechanisms associated with OC [4].

Cellular immunotherapy aims to induce tumor-specific helper and cytotoxic T-cells capable of efficiently targeting and eradicating OC. This has been attempted by in vitro expansion of TIL [5], and engineered T-cells [6]. Nonetheless, the most efficient physiological process for T-cell priming requires full dendritic cell (DC) activation and antigen presentation. One of the major obstacles to the development of effective DC-based immunotherapy in OC is the circumvention of tumor-associated immunosuppressive mechanisms, the most notable being the accumulation of tumor-infiltrating Treg cells, which have been associated with increased mortality in patients [7]. Thus, it is necessary to “program” DC towards activation of effector T-cell polarizing profiles and avoidance of Treg expansion.

Regulation of the p38 and ERK signal transduction pathways in DC plays a central role in determining the expression of certain cytokines by DC. As an example, inhibition of MEK 1/2 and MAPK promotes IL-12 production and Th1-polarizing responses, whereas inhibition of p38 MAPK blocks IL-12 production [8]. Because IL-12 facilitates the differentiation of type 1 T-helper cells, the inhibition of IL-12 secretion in DC is expected to negatively affect DC-driven anti-tumor T-cell responses [9]. Interestingly, p38 inhibition promotes differentiation and survival of monocyte-derived DC [10], and p38 inhibition or ERK activation restores deficiencies in DC function in myeloma patients [11], suggesting that treatment of DCs with pharmacological p38 inhibitors may be therapeutically useful. Furthermore, blockade of the p38 pathway can attenuate regulatory T cell induction by DC [12], whereas blockade of the ERK pathway suppresses DC-driven Th17 responses [13], suggesting that p38 blockade (which enhances ERK phosphorylation) may favor a switch from Treg induction to Th17 differentiation. This could be of critical importance for DC vaccination against OC since Treg expansion and infiltration is known to correlate with increased morbidity and mortality, [7] whereas Th17 infiltration is strongly associated with prolonged patient survival [14].

Cancer/testis antigens (CTA) are a novel class of tumor-associated antigens displaying potent immunogenicity and high expression levels in tumor cells but negligible expression in normal tissues. We have successfully exploited the cancer/testis antigen mSP17 for adoptive immunotherapy in OC [15] and as a biomarker to track OC progression in animal models [16]. The suitability of mSP17 as a target for immunotherapy is supported by the finding that it is expressed by primary and metastatic OC lesions in up to 70% of patients [17]. Further, the relevance of mSP17 expression in OC has been confirmed by the demonstration that it is correlated with malignancy, chemo-resistance and tumor cell motility [17]. We have also shown that recombinant adeno-associated virus vectors (rAAV) can successfully deliver tumor antigens in DC and are superior to protein loading techniques for MHC class I-restricted cellular mediated immunotherapy in multiple myeloma [18], cervical [19] and ovarian cancers [20].

In this study, we combine pharmacological p38 MAPK inhibition with rAAV-based mSP17 antigen expression for autologous DC vaccination in the ID8 mouse model of epithelial OC, a system that faithfully reproduces the peritoneal dissemination seen advanced human OC [21]. We present evidence that mSP17-targeted DC vaccination, following inhibition of p38 MAPK, results in a dramatic enhancement of the therapeutic efficacy of DC vaccination. Finally, we validated our results by demonstrating that p38 blockade in the presence of rAAV Sp17 transduction is superior to rAAV Sp17 transduction alone, in terms of activation of human DC and their ability to generate effector lymphocytes.

## Methods

### Construction of rAAV-mSP17 virus

The AAV-mSP17 genome was constructed as a plasmid as previously described [18, 20]. The mSP17 cDNA was inserted into the rAAV vector, dl6-95 [22]. The mSP17 gene was expressed from the rAAV p5 promoter, which is well described in the literature to be active in DC [22]. We evaluated their ability to infect HEK293 cells using our rAAV-mSP17 construct. The human rAAV-SP17 vector was produced following the same scheme described for mSP17. Human full length SP17 coding sequence was obtained by gene synthesis from GenScript.

### Titration of virus stocks

DNA was extracted from virus crude lysates, and the titer of virus stocks was determined by real-time PCR. Briefly, we used serial dilutions of the corresponding rAAV vector for construction of a standard curve. The real-time PCR was performed on an ABI Prism 7000 instrument (Applied Biosystems, Darmstadt, Germany) in a 50 μl reaction volume.

### Generation of dendritic cells (DC)

Murine DC were generated from splenocytes. We infected adherent monocytes with rAAV as previously described [18, 19, 23]. 10 µM p38 MAPK inhibitor (catalog #ML3403, EMD Chemicals, La Jolla, CA) was added at days 0, 3 and 5. Human mature DC were differentiated from PBMCs of five ovarian cancer patients [24, 25]. rAAV-transduced human DC were differentiated from rAAV-infected monocytes as we have previously described [18, 19]. 10^7^ rAAV-SP17 encapsidated genomes (eg) were used to transduce adherent monocytes in 6-well plates [26]. To block p38, we used the same schedule and concentration detailed above for murine cells.

### Mice and ID8 cell line

Six-week-old female C57BL/6 mice were obtained from the Jackson Laboratory (Bar Harbor, ME, USA). Approval for the study was obtained from the local Institutional Animal Care & Use Committee. The ID8 cell line was kindly provided by Dr. Roby (University of Kansas). Cells were cultured in RPMI 1640 medium supplemented with 10% fetal bovine serum in 5% CO_2_ at 37 ℃.

### Immunization and tumor challenge

Female C57BL/6 mice (6 weeks old) were challenged i.p. with 1 × 10^6^ ID8 cells [1]. 30 days after tumor challenge, mice were i.m. injected once a month for 10 months with rAAV or rAAV-mSP17 transduced DC treated or not with p38 MAPK inhibitor. Each mouse was injected with 10^6^ DC.

### ELISpot assay

Cytokine expression by splenocytes challenged in vitro with 1 ug/mL recombinant SP17 protein or irrelevant protein (BSA) was evaluated using an ELISPOT assay (U-CyTech, Utrecht, Netherlands), according to the manufacturer’s directions. Positive control for cellular activation was Con-A (5 µg/mL), and background wells contained RPMI 1640 medium only. Spot counts were performed with the AID ELISPOT Reader System (Cell Technology, Inc., MD). Results represent the average number of spots in each condition obtained with SP17-challenged splenocytes negative the number of spots obtained with BSA-challenged solenocytes (no spots were detected in the background control).

### Cytotoxicity assay

We performed a EUROPIUM-based cytotoxicity assay using the DELFIA^®^ EuTDA system according to the manufacturer’s instructions [27] with DC-co-cultured splenocytes. To assay the memory response, we performed an identical assay using unprimed splenocytes taken from mice at the time of necropsy.

### Migration assay

The bottom chambers of polycarbonate Corning^®^ Transwell™ Permeable Supports (5 μm pore size, Cole-Parmer, Vernon Hills, Illinois) were coated with ID8 cells. 200,000 splenocytes were added in the upper chamber in complete medium. After 4 h, the density of migrated cells in the bottom wells was determined. The assay was performed in triplicate and mean ± SEM were determined.

### Flow cytometry

Flow-cytometric analyses were performed with a Beckman-Coulter FC500 flow cytometer and CXP Software. Cells were washed twice in PBS, incubated with 10% autologous serum in PBS for 30 min on ice to block Fc receptors, then incubated with phycoerythrin-conjugated anti-CD3 PE-CF-594 antibody (BD Biosciences) for 1 h on ice in staining buffer (0.5% BSA in PBS). After washing twice in 0.5 mL staining buffer, cells were fixed with 4% buffered paraformaldehyde at 4 C for 30 min in the dark. Then, cells were washed twice with staining buffer and permeabilized with 0.5% saponin in staining buffer (permeabilization buffer) for 10 min on ice, washed once with 0.5 mL permeabilization buffer and resuspended in 50 μL buffer/200,000 cells. Anti-Foxp3-FITC anti-IFNγ Alexafluor 647 (Bio Legend), anti-TNFα Alexafluor 700 (BD Biosciences), were added and allowed to incubate for 1 h on ice. Cells were washed twice with permeabilization buffer (200 µL) and resuspended in 400 µL staining buffer supplemented with 1% W/V paraformaldehyde and stored at 4 C in the dark until data acquisition.

### Preparation of lymphocytes

Briefly, the spleen was meshed with a sterile filter, and then centrifuged at 800×*g* for 5 min at 4 °C. Splenocytes were then isolated by Lympholyte-M (Cedarlane Ltd, Burlington, NC). Human lymphocytes were recovered from the non-adherent fraction of purified PBMCs after 3 h incubation in 6-well plates.

### Generation of human DC-primed lymphocytes

For experiments with human cells, non-adherent PBMCs from the same subject were washed in PBS and resuspended in serum-free DC-medium (CellGenix) at 10^6^ cells/well in 6-well culture plates, with autologous DC (20:1 PBMCs to DC ratio) [19, 24]. Cultures were supplemented with 800 U/mL GM-CSF and 10 U/mL IL-2 for 7 days prior to analysis.

### Statistical analyses

Tumor growth, cytotoxicity assays, ELISPOT, migration assays and flow-cytometry were analyzed by a two-tailed, paired Student’s test and survival rates were analyzed by the log-rank test.

## Results

### Vaccination with rAAV-mSP17 transduced autologous DC treated with p38 MAPK inhibitor provides long-term survival advantage

**Fig. 1:**
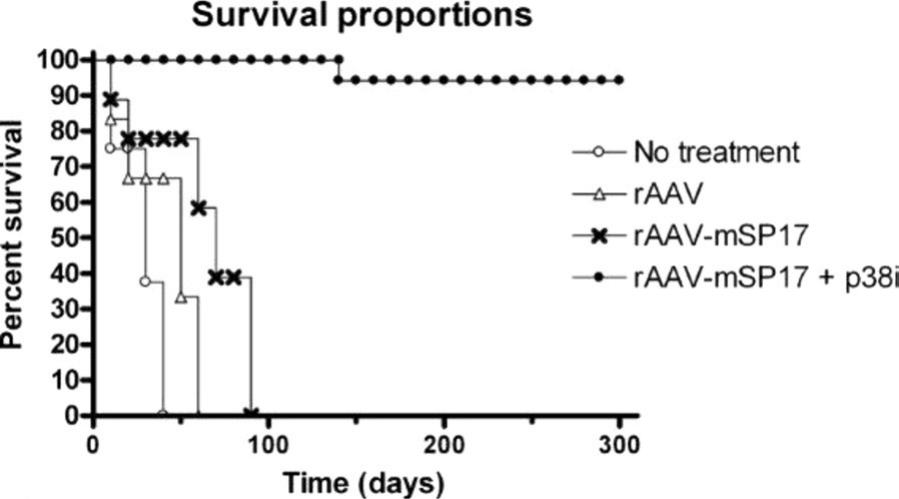
Survival rates of mice that received different DC vaccination protocols. Survival rates are presented as the percentage of live mice in each group per day. Experimental end-point was day 300. Statistically significant survival curves using a log-rank (Mantel-Cox) test (p < 0.0001) are shown

A total of 50 C57BL/6 female mice were included in the study per experiment. 40 mice were i.p. injected with 10^6^ ID8 cells [1] and randomly assigned to the following groups after 30 days: animals in group 1 received i.p. injection with 10^6^ rAAV-mSP17 engineered DC pre-treated with p38 MAPK inhibitor (rAAV-mSP17 DC + p38i), group 2 received 10^6^ rAAV-mSP17 DC without pre-treatment (rAAV-mSP17), group 3 received 10^6^ DC transduced with rAAV vector alone, while group 4 was not treated. Injections were performed every 30 days. Treatment outcomes were assayed through in vivo and in vitro analyses. Two independent experiments were performed. Analysis of survival shows that rAAV-mSP17 DC + p38i vaccine prevents mortality for at least 10 months, representing a dramatic improvement in survival rates compared with tumor-bearing mice vaccinated with rAAV-mSP17 DC or rAAV DC (Fig. 1a). Specifically, 95% of rAAV-mSP17 DC + p38i vaccinated animals survived up to 300 days, while rAAV-mSP17 DC-vaccinated mice or rAAV DC-vaccinated mice died within 98 and 60 days, respectively.

### Th1 cytokines-expressing cells are is elevated in rAAV-mSp17 DC + p38 vaccinated mice

**Fig. 2:**
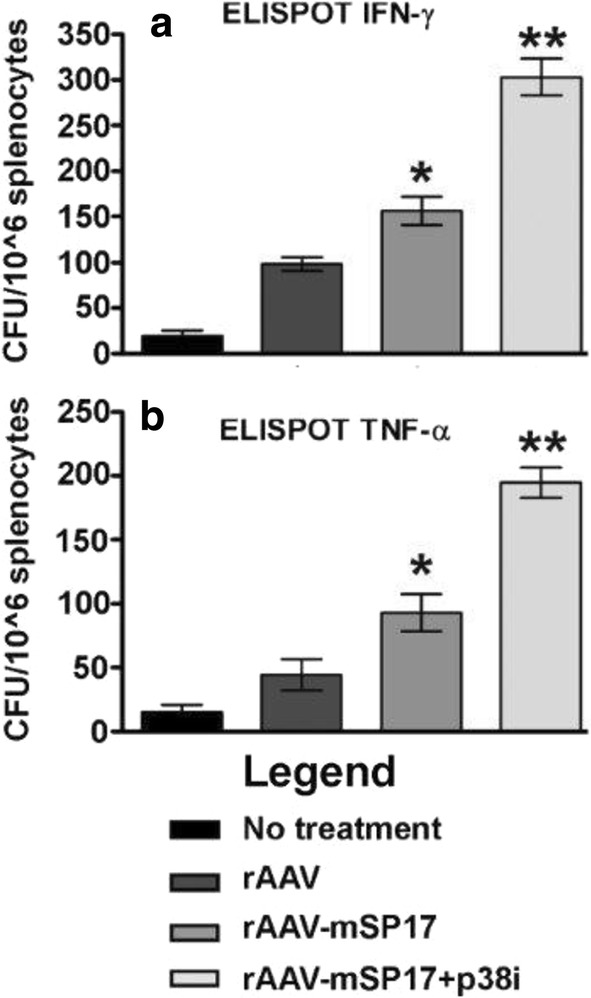
**a**, **b** Measurement of cytokines production following vaccinations. Splenocytes from vaccinated mice and controls (5 animals/group) were collected post-mortem and analyzed by ELI-Spot assay. Data are presented as the frequency of IFN-γ and TNF-α spot-forming cells per 10^6^ splenocytes. Spot numbers represent the mean of ten mice for each vaccination; bars, SD calculated in triplicates. Two-tailed T-test p value versus no treatment group < 0.05 (*) or < 0.01 (**)

EliSPOT assays showed that the frequency of IFN-γ and TNF-α secreting lymphocytes collected from the spleens of rAAV-mSP17 + p38i DC vaccinated mice was significantly higher than that of untreated DC vaccinated mice (Fig. 2a, b respectively).

### rAAV-mSP17 DC + p38i vaccination induces potent cytotoxic responses against ID8 cells

**Fig. 3:**
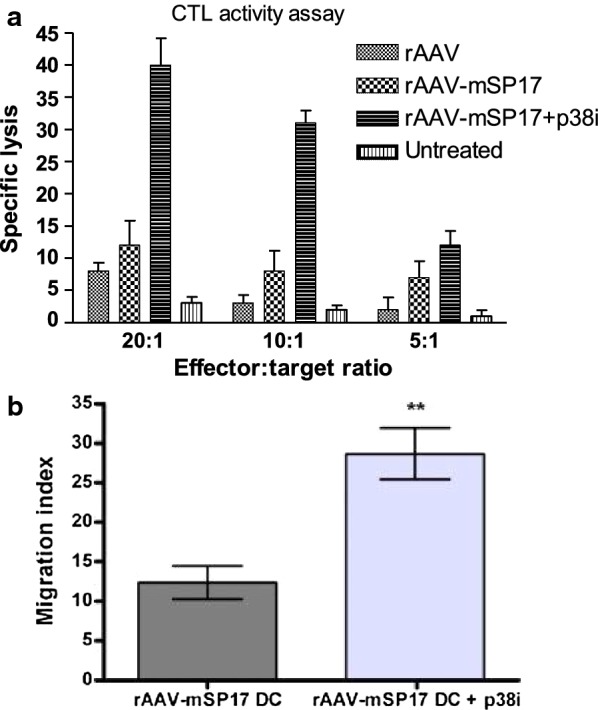
Evaluation of lymphocyte response against ID8 cells in vitro. **a** Cytotoxic lymphocyte responses against ID8 cells following DC vaccination. Anti-tumor cytotoxic activity of in vitro-stimulated splenic lymphocytes was evaluated through a EUROPIUM assay at the indicated effector (E): target (T) ratios. rAAV-mSP17 DC vaccination increased ID8 cell lysis in comparison with rAAV DC (* = two-tailed T-test p = 0.03). Significantly higher rates of lysis were obtained following vaccination with rAAV-mSP17 DC + p38i (** = two-tailed T-test rAAV-mSP17 + p38i versus rAAV-mSP17 or rAAV p < 0.01). **b** DC vaccination promotes lymphocyte migration towards tumor cells. Splenocytes isolated from rAAV-mSP17 + p38i DC vaccinated (rAAV-mSP17 + p38i) or rAAV-DC vaccinated mice (rAAV-DC) were isolated and loaded in the upper chamber of a Transwell chemotaxis plate. The lower chambers were seeded with sub-confluent ID8 cells. After 4 h-incubation at 37 °C and 5% CO_2_, non-adherent cells in the lower chamber were counted and migration indexes were calculated. The histogram shows the migration indexes computed as the ratio between the number of migrated splenocytes in the presence of ID8 cells divided by the number of migrated splenocytes in the presence of culture medium alone (without tumor cells). The assay was run in triplicate and mean values ± SEM are shown (** = two-tailed T-test p < 0.01)

Cytotoxicity assays were performed using ID8 cells as targets and splenocytes taken post-mortem from control or rAAV DC, rAAV-mSP17 DC or rAAV-mSP17 DC + p38i mice after priming with autologous DC as effectors. rAAV-mSP17 DC vaccine was effective in increasing lysis of ID8 cells in comparison with rAAV DC (two-tailed T-test p = 0.03 for E:T ratio 20:1 and 10:1), but significantly higher lysis rates were obtained following vaccination with rAAV-mSP17 DC + p38i (Fig. 3a; two-tailed T-test rAAV-mSP17 + p38i versus rAAV-mSP17 or rAAV p < 0.01 for E:T ratio 20:1 and 10:1).

### rAAV-mSP17 DC + p38i vaccination amplifies splenocytes migration towards ID8 cells

To evaluate the ability of splenocytes to traffic toward tumor cells we used a transwell migration assay. Figure 3b displays migration of splenocytes from mice treated with different vaccine formulations (rAAV-mSP17 DC + p38i or control rAAV-mSP17 DC). In the presence of ID8 cells, we observed a 2.5-fold increase in migrating splenocytes from rAAV-mSP17 DC + p38i vaccinated mice compared with splenocytes from rAAV-mSP17 DC vaccinated mice.

### p38 inhibition improves the activation phenotype and the T cell activation potential of human DC derived from rAAV-transduced monocytes in vitro

**Fig. 4:**
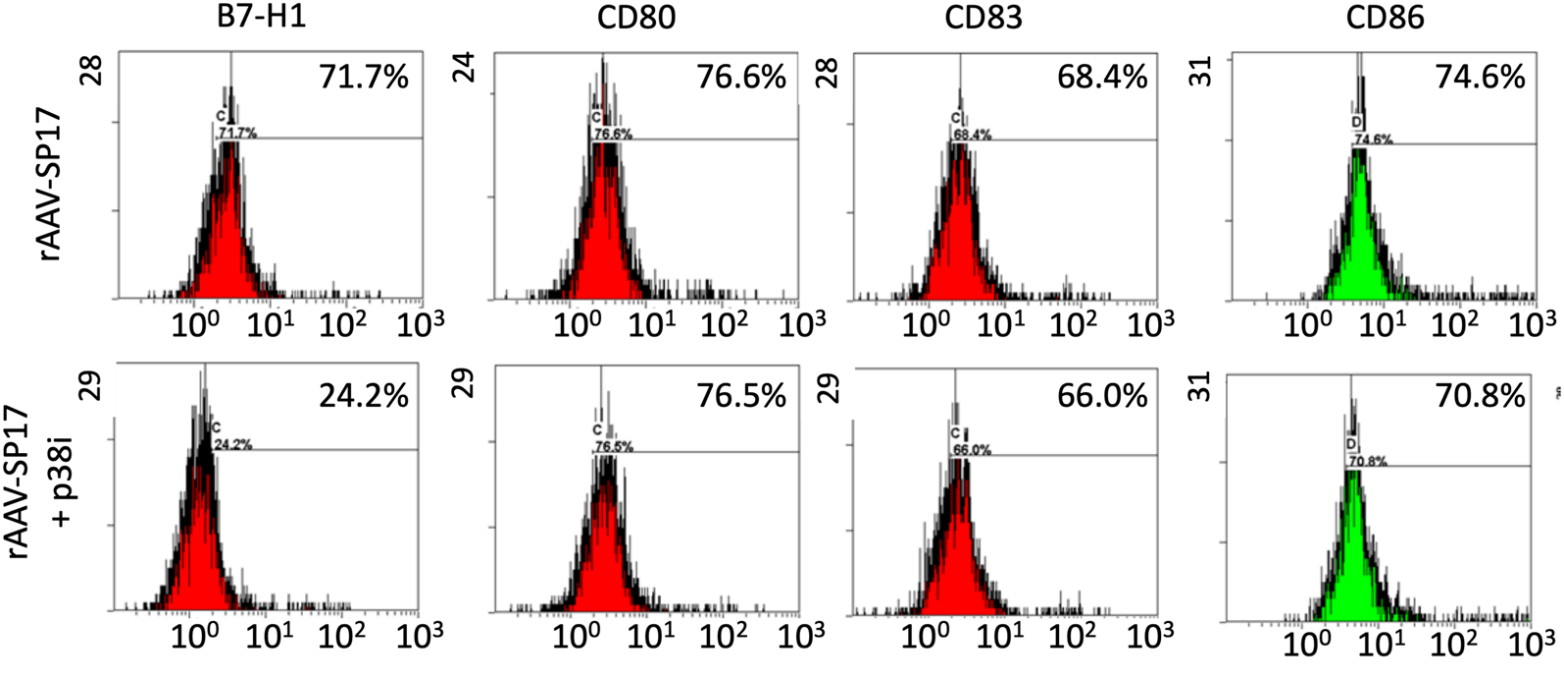
Representative flow-cytometry analysis of human monocyte-derived DC. Histograms from one representative subject are shown. The cut-off was set at the maximum fluorescent intensity level given by the corresponding isotypic control

**Fig. 5:**
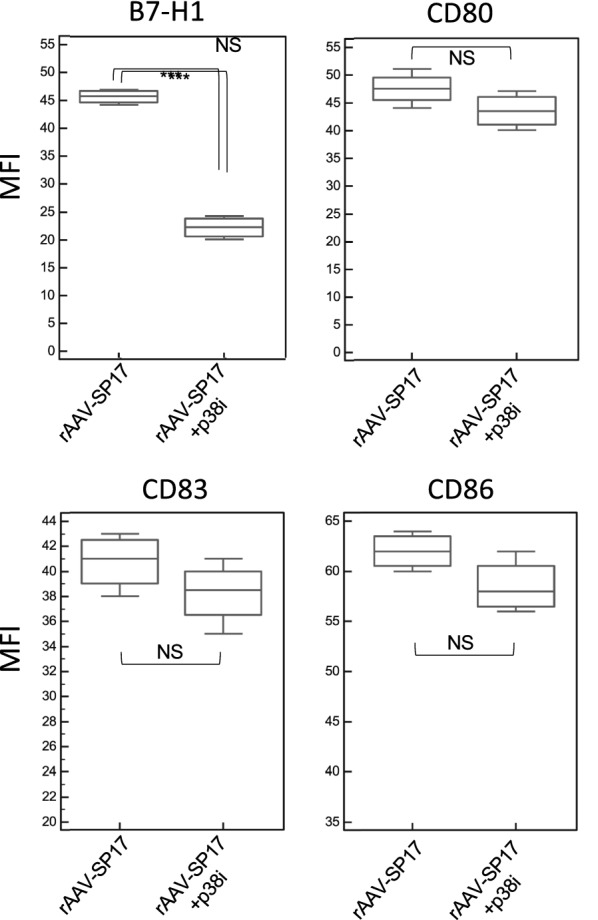
Mean fluorescence intensity (MFI) analysis. The MFI of the indicated markers was recorded out of five experiments. Whiskers represent range of values, boxes represent first and third quartiles, while horizontal lines are the medians. Statistical analysis was performed by a two-tailed T-test (α = 0.05; *** p < 0.001; *NS* not significant)

To evaluate the validity of our results in human cells, human peripheral blood adherent monocytes were used to differentiate DC in vitro, and their activation status was assayed by flow-cytometry for CD80, CD83, CD86, and B7-H1. Figure 4 shows representative histogram plots, while Fig. 5 depicts the MFI values and results of statistical analysis. Two sets of DC were generated, namely rAAV-SP17 transduced DC, and rAAV-SP17 + p38i transduced DC. In the presence of p38i, DC showed statistically significant down-regulation of B7-H1 only, whereas CD80, CD83, and CD86 were unchanged, compared with rAAV-SP17 transduced DC (Figs. 4 and 5).

**Fig. 6:**
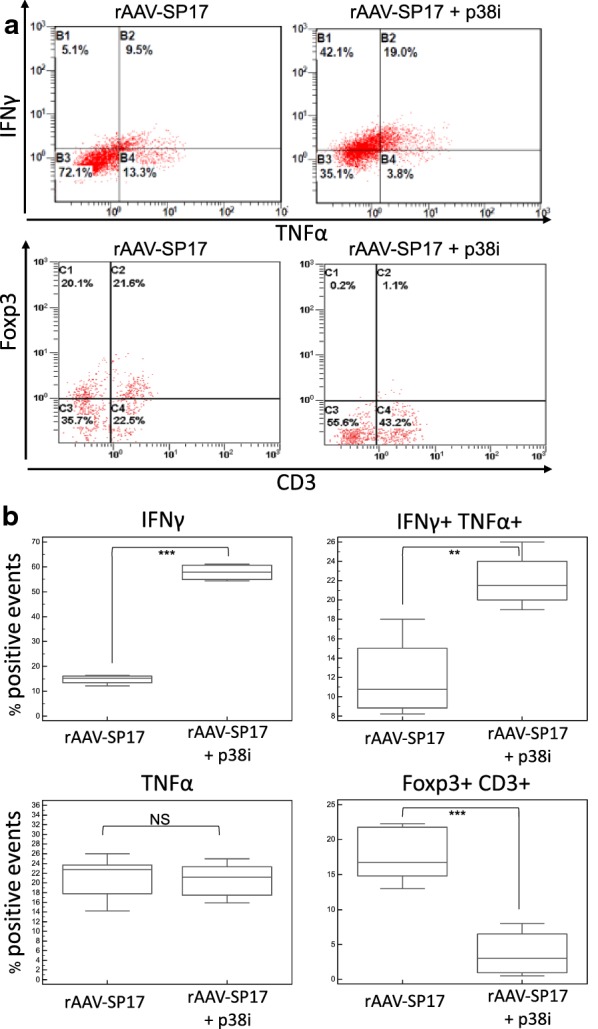
Flow cytometry analysis of DC-primed autologous PBMCs derived from human subjects. **a** Shows the dot-plots of double staining analyses. **b** Summarizes the percentage of positive events recorded out of five experiments and shows the statistical analyses (two-tailed T-test with α = 0.05; *** p < 0.001; ** 0.001 < p<0.01; *NS* not significant)

Non-adherent autologous PBMCs were then co-cultured with different DC preparations detailed above for 7 days, as described in Methods, and their activated versus regulatory phenotype was assayed by flow-cytometry for Foxp3 in the CD3 population (suppressor T cells) and for IFNγ/TNFα (activated effector T cells). Figure 6a shows representative plots from one subject and Fig. 6b shows the statistical analyses. The blockade of p38 did not affect the frequency of TNFα^+^ cells but resulted in an increase of IFNγ^+^ and consequently of TNFα^+^IFNγ^+^ cells (Figs. 6b). Consistently with these findings, p38i significantly reduced the frequency of Foxp3^+^ T cells (Foxp3^+^CD3^+^, Fig. 6b).

## Discussion

OC has been referred to as a “silent killer”, since the lack of specific symptoms prevents detecting the disease in early, ovary-confined stage with favorable prognosis, when nearly 90% of patients could be saved [23]. Therefore, OC remains a lethal disease [28]. Cellular immunotherapy, based on adoptive T-cell or DC transfer, has the potential to provide long-term protection and prevent metastatic dissemination of the tumor [6, 22, 29–31]. In this report, we describe the efficacy of an innovative therapeutic vaccine based on DC treated with a p38 MAP-kinase inhibitor, and transduced with an mSp17 expression rAAV vector.

We and others have reported SP17 as a useful tumor cell biomarker and ideal target for immunotherapy in a variety of malignancies, including multiple myeloma, nervous system tumors, esophageal cancer and OC [15, 16, 21–23, 32–35]. We have shown that the widely used ID8 cell line model of OC expresses significant levels of SP17 protein, both at the cell surface and cytoplasm [1], resembling the majority of primary and metastatic tumor samples obtained from epithelial OC patients [15, 17] and supporting the rationale for SP17-targeted immunotherapy. Further characterization of ID8 cells through flow cytometry revealed that 95% of cells were MHC class I-positive, [1] a relevant finding since antigen presentation within MHC class I complexes is a required step for cytotoxic T-cell target recognition.

Here we tested two different vaccine strategies based on autologous DC transduced with rAAV-mSP17, with or without exposure to a p38 MAPK inhibitor (rAAV-mSP17 DC ± p38i), and compared those with two controls, i.e. rAAV-transduced DC (without mSP17 coding sequence) or absence of vaccination. Our results clearly demonstrate benefits of this innovative DC vaccination. rAAV-mSP17 DC and rAAV-mSP17 DC + p38i vaccines resulted in increased survival rates, but long-term protection was achievable only when p38 MAPK signaling within the DC was blocked. Tumor mass and ascites production were markedly diminished, and survival was extended following rAAV-mSP17 DC administration compared with rAAV DC, but the best protection was provided by the rAAV-mSP17 DC + p38i vaccine, which prolonged survival for at least 300 days. We have previously shown that rAAV-based antigen transduction of DC could induce potent MHC class I-restricted in vitro immune responses against OC and multiple myeloma, overcoming immune tolerance [18, 20]. Here, we extended our study to show that rAAV-transduced DC vaccination provides excellent therapeutic results in vivo. The efficacy of DC vaccination was clearly enhanced through p38 MAPK inhibition, allowing exceptional levels of protection against tumor growth in syngeneic recipients. These observations are highly significant, since long-term protection against OC progression is critical to control fatal disease relapse [36].

We showed that rAAV-mSP17 DC + p38i vaccination significantly increased the frequency of IFN-γ and TNF-α-producing T-cells compared to rAAV-mSP17 DC or AAV DC vaccines. This is an important finding since IFN-γ and TNF-α have been shown to provide protective effects in OC patients [37–39]. These results suggest that p38 MAPK inhibition directs DC differentiation towards a Th1-polarizing profile [40].

Our observations are in accordance with the superior anti-tumor cytotoxic activity displayed by the splenocytes of rAAV-mSP17 + p38 inhibitor treated mice, compared to all of the other groups. Of note, the cytotoxic response was achieved without re-stimulation of splenocytes with autologous dendritic cells in vitro, indicating that our vaccine strategy might have induced a memory response, a hypothesis that is supported by the long (300 days) persistency of the survival advantage seen in the rAAV-mSP17 + p38 inhibitor group.

It is self-evident that stimulation of strong anti-tumor effector T cell responses is of little benefit to the host unless the T cells have the capacity to migrate into the tumor microenvironment. Since lymphocytic tumor infiltration is considered a positive prognostic factor in OC patients, the observation that rAAV-mSP17 DC + p38i vaccine resulted in an increased ability of the host splenocytes to migrate towards ID8 OC cells highlights the potential clinical relevance of our results.

Since T-reg infiltration is associated with increased mortality in OC patients [7], DC vaccination strategies that diminish T-reg activation and expansion and increase Th1 and CTL responses may result in strong anti-tumor immune reactivity and increased survival in OC patients [14, 41]. Recent studies have pointed to p38 MAPK in DC-driven induction of T-reg responses. For example, Jarnicki and colleagues have shown that Toll-like receptor (TLR) activation promotes induction of T-reg through DC production of IL-10 dependent on p38 MAPK signaling [12]. In addition, clinical studies in myeloma have shown that inhibition of p38 MAPK can correct defects in DC function, restoring their ability to activate alloreactive and tumor antigen-specific T cells [11].

Accordingly to our previous report [25], p38 inhibition in differentiated DC affords for a dramatic down-regulation of the T-cell inhibitory signaling molecule, B7-H1. Indeed, we found that p38 inhibition did not significantly alter CD80/83/86 expression levels in autologous human rAAV-Sp17 DC but reduced that of B7-H1. Consistently, when analyzing DC ability to induce activated T cells versus T-reg cells in vitro, we found that non-adherent PBMCs co-cultured with DC differentiated in the presence of p38i displayed both a reduced expression of the T-reg marker Foxp3 and up-regulation of the T cell activation marker, IFNγ. While it is known that rAAV transduction offers advantages over standard DC antigen pulsing methods, in terms of up-regulation of co-stimulatory molecules and consequently of T-cell activation [19, 20] due to the known pro-inflammatory mechanisms triggered by AAV entry [42], the molecular mechanism explaining the different outcome of p38 blockade on B7-H1 and CD80/83/86 remains to be elucidated. Indeed, we have previously demonstrated that p38 inhibition alone, without rAAV, results in a down-regulation of CD80/83/86 expression along with B7-H1 in DC [25]. Our hypothesis is that CD80/83/86 expression in p38i-treated DC in the presence of rAAV is rescued by the virus ability to activate the NF-κB pathway [43], which has been proven to increase the levels of co-stimulatory molecules in DC [44].

## Conclusions

Our results show that rAAV-Sp17 engineered DC vaccination that incorporates p38 inhibition induces long-term protection in a murine model of OC. Our results suggest that the use of this rAAV-Sp17 transduced DC vaccine, together with p38 inhibition, may prove a suitable strategy to generate potent anti-tumor responses in OC patients. Therefore, our findings support the development of early stage clinical trials to determine the safety of rAAV-Sp17 transduced DC vaccination, together with p38 inhibition, and its ability to promote immunity and reduce regulatory T cells in human OC.

## Data Availability

All data generated or analysed during this study are included in this published article.

## Abbreviations

CTL: cytotoxic T cells
DC: dendritic cells
e.g: encapsulated genomes
OC: ovarian cancer
p38i: p38 MAPK inhibitor
PBMCs: peripheral blood mononuclear cells
rAAV: recombinant adeno-associated viral vector
SP17: sperm protein 17
Th: helper T cells
